# The Hartung‐Knapp modification for random‐effects meta‐analysis: A useful refinement but are there any residual concerns?

**DOI:** 10.1002/sim.7411

**Published:** 2017-07-26

**Authors:** Dan Jackson, Martin Law, Gerta Rücker, Guido Schwarzer

**Affiliations:** ^1^ MRC Biostatistics Unit Cambridge UK; ^2^ University of Freiburg Germany

**Keywords:** random‐effects models, small sample inference, statistical conventions

## Abstract

The modified method for random‐effects meta‐analysis, usually attributed to Hartung and Knapp and also proposed by Sidik and Jonkman, is easy to implement and is becoming advocated for general use. Here, we examine a range of potential concerns about the widespread adoption of this method. Motivated by these issues, a variety of different conventions can be adopted when using the modified method in practice. We describe and investigate the use of a variety of these conventions using a new taxonomy of meta‐analysis datasets. We conclude that the Hartung and Knapp modification may be a suitable replacement for the standard method. Despite this, analysts who advocate the modified method should be ready to defend its use against the possible objections to it that we present. We further recommend that the results from more conventional approaches should be used as sensitivity analyses when using the modified method. It has previously been suggested that a common‐effect analysis should be used for this purpose but we suggest amending this recommendation and argue that a standard random‐effects analysis should be used instead.

## 1 INTRODUCTION

The random‐effects model for meta‐analysis^1^, ^2^ is now widely used to pool heterogeneous estimates and also obtain a meaningful estimate of the average effect. The random‐effects model provides a way to relax the common‐effect (also often referred to as the fixed‐effect) model's usually implausible assumption that the studies estimate the same true underlying effect. The standard method for making inferences using the random‐effects model goes back at least as far as the seminal paper by DerSimonian and Laird.^3^

The main statistical difficulty when applying the random‐effects model in practice is that we must estimate the between‐study variance. The precision of the estimated between‐study variance is generally low in small samples (ie, meta‐analyses with few studies), and this is reflected in the often very wide confidence intervals for this parameter.^4^ The uncertainty in this estimate is not taken into account when applying the random‐effects model to make inferences about the average effect in the usual way, which means that the resulting statistical inference is often not very accurate. For example, the actual coverage probability of confidence intervals for the average effect can deviate markedly from the nominal probability in small samples.^5^ In particular, when substantial heterogeneity is present, the standard method produces actual coverage probabilities that are substantially less than the nominal level. Wider confidence intervals are needed in such instances to reflect the uncertainty in the between‐study variance and so more satisfactorily provide the nominal coverage probability.

In an attempt to improve upon the repeated sampling properties of the standard method for random‐effects meta‐analysis, Hartung and Knapp^6^, ^7^, ^8^ and Sidik and Jonkman^9^ propose a modified (or refined) method. Like the more conventional and now standard method, their modified method involves estimating the between‐study variance and then treating this as if fixed and known. Hence, both the standard and the modified methods are approximate methods. However, further calculations are made by the modified method which justify using quantiles from a *t*, rather than a standard normal, distribution for making inferences about the average effect. Although questions have been raised about the validity of the modified method,^10^, ^11^ this method has become accepted and advocated for general use.^12^, ^13^ Furthermore, the modified method has been implemented as an option in some standard meta‐analysis software, for example, in the *metafor* 14 and the *meta* 15 packages in *R*. Extensions of the modified method for meta‐regression^16^ and multivariate meta‐analysis^17^ have also been proposed.

Although alternative ideas have been suggested for improving the performance of frequentist methods for random‐effects meta‐analysis,^18^, ^19^ the modified method is appealing because it introduces no further numerical or computational difficulties: if noniterative estimates of the between‐study variance are used, or iterative methods are used that ensure convergence, then both the standard and the modified methods do not encounter numerical problems. Furthermore, the theory that underlies the modified method is relatively simple and is closely related to methods used in particle physics.^20^, ^21^ Part of the attraction of the modified method therefore must be that it is intuitively appealing. Simulation studies^7^, ^8^, ^9^, ^12^ reassure us that the modified method provides actual coverage probabilities that are closer to the nominal level than the standard method.

Given all the advantages of the modified method, it is perhaps surprising that it has not been more widely adopted. In this paper, we will explore the issues presented by the modified method, and in doing so, we will carefully assess possible explanations for why it is not used more often in application. The rest of the paper is set out as follows. In Section 2, we briefly describe both the standard and modified methods for random‐effects meta‐analysis. In Section 3, we discuss a variety of possible concerns faced by those who would advocate the modified method. In Section 4, we describe some different conventions that can be adopted when using the modified method. In Section 5, we present our new taxonomy of meta‐analysis datasets that we use as a framework to assess the consequences of these conventions. To investigate the implications of using the proposed conventions in practice, in Section 6, we perform an empirical investigation. We conclude in Section 7 with a short discussion.

## 2 THE STANDARD AND MODIFIED METHODS FOR RANDOM‐EFFECTS META‐ANALYSIS

The conventional random‐effects model assumes that the estimate from the *i*th study, *i*=1,2,⋯*n*, is distributed as $Y_{i}∼N(\mu ,\sigma_{i}^{2}+\tau^{2})$. The within‐study variances $\sigma_{i}^{2}$ are treated as fixed and known in analysis but are estimated in practice. The parameter *μ* is the average effect and is the parameter of primary interest; *τ* ^2^ is the between‐study variance. In the first computational stage, *τ* ^2^ is estimated. This estimate can be obtained in a variety of ways,^22^ which provides variants of both the conventional and modified methods. If ${\hat{\tau }}^{2}=0$, then the random‐effects model is said to collapse to the common‐effect model (where *τ* ^2^ is assumed to be 0). Inferences for the average effect are made in the second computational stage. The estimated average effect is $\hat{\mu }=\sum w_{i}y_{i}/\sum w_{i}$, where $w_{i}=1/(\sigma_{i}^{2}+{\hat{\tau }}^{2})$. The same point estimates are obtained using the standard and modified methods under the random‐effects model.

Confidence intervals and hypothesis tests immediately follow using the standard method by assuming that

(1) $$\left(\hat{\mu }-\mu )\sqrt{\sum w_{i}}∼N(0,1\right)$$

so that, for example, a 95% confidence interval is obtained as $\hat{\mu }\;\pm \;1.96/\sqrt{\sum w_{i}}$.

The modified method involves further calculating a quadratic form, whose square root we will call *H* ^∗^, where

(2) $$H^{*2}=\frac{\sum w_{i}{\left(Y_{i}-\hat{\mu }\right)}^{2}}{n-1}.$$

If ${\hat{\tau }}^{2}=0$, then *H* ^∗^=*H*, where *H* is the square root of the *H* ^2^ heterogeneity statistic proposed by Higgins and Thompson. 23 If all variances (both within and between studies) are taken as known, then confidence intervals and hypothesis tests immediately follow using the modified method by assuming that

(3) $$\frac{(\hat{\mu }-\mu )\sqrt{\sum w_{i}}}{H^{*}}∼t_{\left(n-1\right)},$$

where *t*_(*n*−1)_ denotes a *t* distribution with (*n*−1) degrees of freedom so that a 100×(1−*α*)% confidence interval is obtained as $\hat{\mu }\pm t_{\left(n-1,\alpha /2\right)}H^{*}/\sqrt{\sum w_{i}}$, where *t*_(*n*−1,*α*/2)_ is the 1−*α*/2 quantile of a *t*_(*n*−1)_ distribution.

Comparing (1) and (3), we can see that the modified method applies a scale factor of *H* ^∗^ to the conventional standard error $1/\sqrt{\sum w_{i}}$ and uses quantiles from *t*_(*n*−1)_ instead of a standard normal distribution. We will therefore refer to $H^{*}/\sqrt{\sum w_{i}}$ as the modified method's standard error so that the modified method can be interpreted as using a different standard error and reference distribution. However, the standard and modified methods result from different types of statistical approximations shown in (1) and (3) and so are perhaps most naturally conceptualised as being completely alternative statistical methods, where no direct reference is made to scaling factors and reference distributions when comparing the 2 methods. We however find the notion of the scaling factor *H* ^∗^, and the use of alternative reference distributions, to be useful ways of describing how the standard and modified methods relate to each other numerically.

To summarise this discussion, the standard and modified methods are conceptually and computationally similar and both can be applied when ${\hat{\tau }}^{2}=0$. In the next section, we will explore some possible reasons why the modified method has not become more widely used in application.

## 3 THE MODIFIED METHOD: 4 CONCERNS

As explained in the introduction, simulation studies have found the modified method to perform better than the standard method. It is therefore natural to ask the question “Why has the modified method not replaced the standard method already?” Part of the reason may be that the standard method is the default in most statistical software and is so commonly used in application that the case for any new, and so less familiar, method must be especially strong for it to be adopted. However, the modified method raises immediate concerns that are not related to any difficulties that might be associated with overcoming statistical inertia. To some extent, we play “devils' advocate” in this section and present 4 potential concerns about the use of the modified method, and so for now, we present the case against it. These concerns are speculative in nature and provide a framework for the material that follows. We will see that some of these concerns, which are very closely related, are not entirely substantiated.

### 3.1 Concern 1: the scaling factor H ^∗^ is a poorly understood random variable

We need to much better understand the distributions of *H* ^∗^, and $H^{*}/\sqrt{\sum w_{i}}$, before we can be comfortable using the modified method. The scaling factor *H* ^∗^, which is applied to the standard error of the pooled effect by the modified method, is a function of the *Y*_*i*_. Hence, it is a random variable. Furthermore, the distribution of the scaling factor *H* ^∗^ is complicated and poorly understood. This is because the *χ* ^2^ distributional assumption for *H* ^∗2^, which leads to the *t*_(*n*−1)_ distribution in (3), is correct under the random‐effects model if $w_{i}=1/(\sigma_{i}^{2}+\tau^{2})$.^24^, ^25^ However, in these equations, we have $w_{i}=1/(\sigma_{i}^{2}+{\hat{\tau }}^{2})$, which distorts the distributional assumptions.^10^ This is because the random‐effect model's “weights,” $w_{i}=1/(\sigma_{i}^{2}+{\hat{\tau }}^{2})$, are also random variables even under the random‐effects model where we take the $\sigma_{i}^{2}$ as fixed and known (because ${\hat{\tau }}^{2}$ is an estimate). The same comments and concerns apply to the distribution of the modified method's standard error, $H^{*}/\sqrt{\sum w_{i}}$. There is also a related concern that taking the within‐study variances as fixed and known can also distort distributional results of this type.^26^ In small samples, the impact that the modification has on the conventional standard error can be anticipated to differ greatly from one meta‐analysis to the next. This means that the implications of using the scaling factor *H* ^∗^ for any given meta‐analysis are hard to predict.

### 3.2 Concern 2: the modified method can result in shorter confidence intervals for the average effect than standard methods

When it is applied to some datasets, the modified method results in shorter confidence intervals, and so smaller *P* values, for the average effect than the standard method. This can occur when *H* ^∗^ is less than 1. The modified method then loses some face validity, because usually the most pressing concern is that the standard method does not take the uncertainty in the between‐study heterogeneity into account and so produces confidence intervals that are too short, resulting in coverage probabilities that are less than the nominal level. Furthermore, as Wiksten et al^27^ point out, the Hartung‐Knapp method is not always conservative compared to a common‐effect meta‐analysis. This is a more serious concern because the common‐effect model makes the strongest assumption about the homogeneity of the data.

A “hardcore frequentist perspective” might be to dismiss the concerns raised by Wiksten et al^27^ as unimportant, irrelevant, or misguided. This is because it is the coverage probability of a confidence interval alone that is its defining characteristic. Hence, some may be satisfied if the nominal coverage probability is (approximately) retained by a confidence interval regardless of any other consideration. Furthermore, a confidence interval that provides short confidence intervals with the correct coverage is likely to be considered highly desirable. In practice, however, we suggest that it is also desirable that confidence intervals possess additional good properties. For example, ideally, they should contain point estimates and be of nonzero but finite length. In the context of random‐effects meta‐analysis, a highly desirable property is that a confidence interval for the average effect should always be at least as wide as the corresponding confidence interval from the common‐effect model. The standard method ensures this, but the modified method does not.

### 3.3 Concern 3: Is it appropriate to use the modified method when ${\hat{\tau }}^{2}=0$?

This type of concern is exemplified by the investigation of Wiksten et al,^27^ who prove that the scaling factor *H* ^∗^ is necessarily less than 1 when the DerSimonian and Laird^3^ estimator is used and ${\hat{\tau }}^{2}$ is truncated to 0. This is a common occurrence in practice.^28^ This would seem to suggest that when ${\hat{\tau }}^{2}=0$, we may be at high risk of producing modified results that are subject to the concerns in Section 3.2 above. Although the theoretical analysis of Wiksten et al relies upon the use of the DerSimonian and Laird estimator (other estimators^22^ have been proposed and can be used in both the standard and modified methods, and we consider 2 alternatives below), we consider their analysis to be instructive for meta‐analyses using other estimators.

There is a more subtle, but very closely related, difficulty for those who would advocate the use of the modified method when ${\hat{\tau }}^{2}=0$. A potential criticism would then be that *given that the estimated between‐study variance is zero, as it is here*, the analysis of Wiksten et al^27^ suggests that the coverage probability of the modified method is not likely to be acceptable (because *H* ^∗^ is necessarily less than one), and hence, it should not have been used in this instance. The difficulty is that the modified method is usually advocated on the grounds of its good unconditional coverage probability, but this concern is based on a concern about a conditional coverage probability. A full discussion of conditional inference is beyond the scope of this paper, but Casella 29 provides an accessible overview and many useful references. As Casella explains, conditional inference relies on identifying a recognisable subset of the sample space (such as the subset of meta‐analyses where the estimated between‐study variance is zero). As Casella also explains, the unconditional coverage probability and the conditional probability, given that the sample lies in the recognisable subset of the sample space, in general differ. Furthermore, “Poor conditional (post data) performance on a recognisable set is taken as criticism” and “only reasonable procedures are free of conditional defects.”^29^ Hence, this potential concern has a sound statistical basis.

### 3.4 Concern 4: The modified method raises the question of whether we should attempt to further modify the standard method

To partially avoid the concern about the considerable variability of *H* ^∗^ in small samples (see Section 3.1), a further ad hoc modification of constraining *H* ^∗^⩾1 has been suggested.^16^, ^17^ This means that the modified method's use of quantiles from the *t* distribution must result in wider confidence intervals than the standard method that uses quantiles from the standard normal distribution. However, this approach can be anticipated to result in overcoverage and hence an inevitable loss of power.

Further modifications are of course possible. Hence, a concern is, by accepting the modified method, we open up possibilities of further ad hoc modifications that are also likely to be objectionable for some reason or another. Thus, rather than substantially improving upon the standard method, the modified method is likely to result in an even wider array of methods and hence confusion for applied analysts.

## 4 THE MODIFIED METHOD: 6 POSSIBLE METHODS OF ANALYSIS

In this section, we describe some methods of analysis that are based on the modified method. As we will explain, some of these possibilities would seem to be motivated by the concerns discussed in Section 3.

### 4.1 Never use the modified method

Although it cannot be truly represented as being an approach that uses the modified method, if we refrain from using the modified method altogether, then we retain the conventional use of the standard method (see Equation (1)). We will therefore present results using the standard random‐effects approach, to compare the other possibilities to.

### 4.2 Always use the modified method

The most obvious and direct use of the modified method is to always use it (see Equation 3). This has been advocated^12^ but is open to the possible concerns described above in Section 3.

### 4.3 Constrain the scaling factor H ^∗^⩾1

Some accounts of the modified method discuss the ad hoc additional modification of constraining the scaling factor *H* ^∗^⩾1. 16, 17 This means that the modified method always results in a more conservative analysis (wider confidence interval) than a standard random‐effects meta‐analysis. This is because if the scaling factor *H* ^∗^ is set to 1, then the use of quantiles from a *t* distribution in the modified method necessarily results in wider confidence intervals than those from the standard method. This convention directly avoids concern 2, and may have been motivated by this and also concern 1, but can be anticipated to come at a price of producing analyses that are overly conservative and so incur a loss of power.

We next describe 3 “hybrid methods” that combine the use of the modified and standard methods, to make use of the modified method and yet also avoid some of the possible concerns in Section 3.

### 4.4 Hybrid method 1: Use the modified method if and only if ${\hat{\tau }}^{2}>0$

This hybrid method is motivated by concern 3, which it directly avoids. Similarly to the previous convention, this is a further modification and so is subject to concern 4.

### 4.5 Hybrid method 2: Apply both the modified and the standard random‐effects method and present the more conservative analysis

Another way to avoid some of the concerns described in Section 3 is to apply both the standard random‐effects and the modified methods and present the more conservative (widest confidence interval) analysis. This convention and all those above provide the random‐effects model's estimate of the average effect $\hat{\mu }$. This convention can however be anticipated to come at a price of producing analyses that are overly conservative and so incur a loss of power. Whether or not the modified method results in a conservative analysis may depend on the value of *α*; we assume that *α*=0.05 has been used throughout. This observation also applies in Section 4.6.

### 4.6 Hybrid method 3: Use both the modified and the standard common‐effect method and present the more conservative analysis

This convention is motivated by the suggestion of Wiksten et al^27^ who suggest presenting a sensitivity analysis using the common‐effect model. By “the more conservative analysis,” we will continue to mean the analysis that provides the wider confidence interval (rather than necessarily provide the larger *P* value; since the common‐effect model estimate of *μ* is not in general equal to the corresponding random‐effect model estimate, it is possible for the random‐effect model to simultaneously result in a wider confidence interval and a smaller *P* value^30^).

These 6 methods are closely related, and some result in the same statistical inferences for particular types of meta‐analysis datasets. To understand the implications of these methods, and also how they relate to each other, we will now develop a new conceptual framework that can be used to compare and contrast the 6 analysis methods.

## 5 A TAXONOMY OF META‐ANALYSIS DATASETS

To provide a framework in which the consequences of the 6 methods described in Section 4 can be assessed, in this section, we present a new taxonomy of meta‐analysis datasets. This taxonomy is sufficient to categorise the type of analysis that each of the 6 methods in Section 4 provide. The taxonomy is designed to serve as both a description of the implications of each of the analysis methods and also as a tool to aid decision making for an “adaptive meta‐analysis,” where decisions regarding which analysis to present as primary depends on the nature of the data.

### 5.1 Motivating the taxonomy

From the concerns in Section 3 and the analysis methods in Section 4, the consideration of 4 criteria is clearly important when determining the implications of using the modified method. The first of these criteria is whether or not ${\hat{\tau }}^{2}>0$(this criterion is directly motivated by concern 3 and hybrid method 1). However, the random variable *H* ^∗^ plays the most direct role in determining the implications of using the modified method, and we should also consider the properties of *H* ^∗^ directly (due to concern 1). An important property of *H* ^∗^ is whether or not it is more than 1, because this determines whether or not the modified method's standard error is greater than the usual one. Hence, the second of our criteria is whether or not *H* ^∗^>1. However, these 2 criteria are not sufficient to fully consider concern 2, because the use of quantiles from a *t*_(*n*−1)_ distribution by the modified methods may overcome the tendency for *H* ^∗^<1 to result in shorter confidence intervals than standard methods. Hence, the third and fourth criteria are whether or not the modified method results in wider 95% confidence intervals than the standard random‐effects analysis and the common‐effect analysis, respectively.

### 5.2 Sixteen possible groups

Our taxonomy is therefore based on 4 binary outcomes so that there are a maximum 2^4^=16 groups of meta‐analyses that comprise it. Specifically, these binary outcomes are (1) ${\hat{\tau }}^{2}>0$, (2) *H* ^∗^>1, (3) the modified 95% confidence interval is wider than the corresponding standard random‐effects model's confidence interval (as mentioned in Section 4.5, this outcome may depend on the value of *α*; this comment also applies to the following fourth outcome and we assume *α*=0.05 throughout), and (4) the modified 95% confidence interval is wider than the corresponding common‐effect model's confidence interval. Our taxonomy was formed by initially assuming that all 16 groups are possible. Groups 1 to 8 were allocated ${\hat{\tau }}^{2}>0$, and groups 9 to 16 were allocated ${\hat{\tau }}^{2}=0$; groups 1 to 4 and 9 to 12 were allocated *H* ^∗^>1, and groups 5 to 8 and groups 13 to 16 were allocated *H* ^∗^⩽1; groups 1 to 2, 5 to 6, 9 to 10, and 13 to 14 were allocated “modified confidence interval is wider than the standard confidence interval” and groups 3 to 4, 7 to 8, 11 to 12, and 15 to 16 were allocated “modified confidence interval is not wider than the standard confidence interval”; finally, odd numbered groups were allocated “modified confidence interval is wider than the common‐effect model confidence interval,” and even numbered groups were allocated “modified confidence interval is not wider than the common‐effect model confidence interval.”

### 5.3 Eliminating 9 impossible groups and the resulting 7 group taxonomy

However, 9 of these 16 groups are impossible to observe, regardless of the estimator of *τ* ^2^ used (some groups can be deemed impossible for multiple reasons). Groups 2, 6, 10, and 14 are impossible because they require the modified method to give confidence intervals that are wider than those from the standard random‐effects method, and yet not wider than those from the common‐effect model. Groups 3, 4, 11, and 12 are impossible because they require both *H* ^∗^>1, and also the modified confidence interval to be not wider than the standard one. Finally, group 15 is impossible because it requires the modified method's confidence interval to be not wider than the standard one, and yet wider than the common‐effect model's confidence interval, when ${\hat{\tau }}^{2}=0$.

The resulting 7 group taxonomy is shown in Table 1. Here, we show the 7 possible groups and also the type of analysis that each of the 6 methods described in Section 4 provides. “Mod” means that the modified standard error $H^{*}/\sqrt{\sum w_{i}}$ is used, “RE” means that the conventional standard error $1/\sqrt{\sum w_{i}}$ is used, and “CE” means that the conventional common‐effect standard error $\sqrt{1/\sum \sigma_{i}^{-2}}$ is used, either because the common‐effect analysis was chosen or because ${\hat{\tau }}^{2}=0$ and the conventional random‐effects analysis collapsed to a common‐effect analysis. *Z* and *t* in parentheses indicate that quantiles from a standard normal or the t distribution are used, respectively.

**Table 1:** A taxonomy of meta‐analysis datasets

					Never	Always	Constrain	Hybrid 1	Hybrid 2	Hybrid 3
Group	${\hat{\tau }}^{2}>0$	*H* ^∗^>1	Mod>RE	Mod>CE	(Section 4.1)	(Section 4.2)	(Section 4.3)	(Section 4.4)	(Section 4.5)	(Section 4.6)
1	Yes	Yes	Yes	Yes	RE(Z)	Mod(t)	Mod(t)	Mod(t)	Mod(t)	Mod(t)
5	Yes	No	Yes	Yes	RE(Z)	Mod(t)	RE(t)	Mod(t)	Mod(t)	Mod(t)
7	Yes	No	No	Yes	RE(Z)	Mod(t)	RE(t)	Mod(t)	RE(Z)	Mod(t)
8	Yes	No	No	No	RE(Z)	Mod(t)	RE(t)	Mod(t)	RE(Z)	CE(Z)
9	No	Yes	Yes	Yes	CE(Z)	Mod(t)	Mod(t)	CE(Z)	Mod(t)	Mod(t)
13	No	No	Yes	Yes	CE(Z)	Mod(t)	CE(t)	CE(Z)	Mod(t)	Mod(t)
16	No	No	No	No	CE(Z)	Mod(t)	CE(t)	CE(Z)	CE(Z)	CE(Z)

As explained in Section 5.3, nine groups are impossible. “Mod>RE” and “Mod>CE” indicate that the modified 95% confidence interval is wider than the corresponding standard random‐effects confidence interval and the corresponding common‐effect interval, respectively. “Mod,” “RE,” and “CE” indicate that the convention results in presenting the confidence interval using the standard error from the modified method, the standard method, and the common‐effect analysis respectively; “(Z)” and “(t)” denote that quantiles from the standard normal and t distribution are used, respectively. For example, “Mod(t)” denotes that the modified standard error and quantiles from a t distribution are used, and “CE(Z)” denotes that the common‐effect standard error and quantiles from a normal distribution are used. For groups 9, 13, and 16, we have ${\hat{\tau }}^{2}=0$ so that “RE” is equivalent to “CE,” but the resulting analysis is represented as “CE.”

Although Table 1 summarises the implications of using each of the conventions for analysis for different types of meta‐analysis datasets, it does not give an indication of which groups are more likely to be observed. Hence, this table is insufficient to fully describe these implications. Our empirical investigation will explore this issue, but some observations from Table 1 are immediate. Firstly, constraining *H* ^∗^⩾1 results in a different analysis to all other conventions unless the meta‐analysis is in group 1 or 9. This shows that, in addition to our intuition that constraining *H* ^∗^⩾1 will be overly conservative, in the majority of groups, this mode of analysis results in a different analysis to other methods. Also from Table 1, we can see that hybrid method 2 is the only non‐standard method that can result in the standard inference (denoted as “RE(Z)”) from the random‐effects model when ${\hat{\tau }}^{2}>0$. However, this only occurs for meta‐analyses that belong to groups 7 or 8, and we will see that these groups are quite rare.

To investigate the implications of each analysis method further, we will investigate how the taxonomy simplifies for particular estimators of *τ* ^2^ and 2 special situations. As we will see, by considering special cases, we can further reduce the taxonomy, and so more easily assess the implications of each analysis method.

### 5.4 Reduced taxonomies for particular estimators

Some further groups in Table 1 are impossible for particular estimators of *τ* ^2^ and so immediately simplify the taxonomy. Many estimators of *τ* ^2^ have been proposed,^22^ and we will restrict our investigation to the Restricted Maximum Likelihood (REML), DerSimonian and Laird, and the Paule‐Mandel estimators because these 3 estimators are especially widely used in application or advocated for this purpose. Hence, we assume throughout that one of these 3 estimators is used.

#### 5.4.1 The REML estimator

We will see in our empirical investigation that all 7 groups in Table 1 are possible when using the REML estimator. Hence, no further reduction in the taxonomy is possible when using REML.

#### 5.4.2 The DerSimonian and Laird estimator

If however the DerSimonian and Laird estimator is used, then group 9 is impossible because it requires ${\hat{\tau }}^{2}=0$ and *H* ^∗^>1 and this is not possible.^27^ All other groups are possible.

#### 5.4.3 The Paule‐Mandel estimator

The Paule‐Mandel estimator^31^ of *τ* ^2^ is defined as the value that satisfies *H* ^∗2^=1. Hence, when a positive ${\hat{\tau }}^{2}$ can be found as the solution to *H* ^∗2^=1, we then have *H* ^∗^=1 by construction. The distinction between groups 1 and 5 is therefore meaningless; the choice between using the criterion of *H* ^∗^>1 or alternatively *H* ^∗^⩾1 in the taxonomy is intended to be of no importance because it was assumed that *H* ^∗^ would be a continuous random variable. Groups 7 and 8 in Table 1 are therefore impossible, because *H* ^∗^=1 means that the modified method must result in wider confidence intervals because of its use of quantiles from a *t* distribution.

Furthermore, if ${\hat{\tau }}^{2}=0$ provides *H* ^∗2^<1 so that there is then no solution for *H* ^∗2^=1, the Paule‐Mandel estimator is truncated to 0. Hence, when the Paule‐Mandel estimator is truncated to 0, then we have *H* ^∗^<1 by construction, and group 9 is impossible. The Paule‐Mandel estimator therefore results in an especially simple 3‐group taxonomy. Assuming that ${\hat{\tau }}^{2}>0$ and the Paule‐Mandel estimator is used, the modified method therefore simply replaces the standard normal quantile with one from a *t* distribution, but otherwise the analysis proceeds as usual.

To obtain the very simple 3‐group Paule‐Mandel taxonomy for all 3 estimators of *τ* ^2^, and so more easily examine the implications of using the modified method, we now consider 2 simple special cases.

### 5.5 The reduced taxonomy for the special case where n=2

When ${\hat{\tau }}^{2}>0$ and *n*=2, we have *H* ^∗^=1 for all 3 estimators of *τ* ^2^. This is partly due to the result of Rukhin,^32^ which states that when *n*=2, the DerSimonian and Laird and REML estimators of *τ* ^2^ coincide and can be written in our notation, and before truncation, as ${\hat{\tau }}^{2}=({\left(y_{2}-y_{1}\right)}^{2}-\sigma_{1}^{2}-\sigma_{2}^{2})/2$. We will see immediately below that the Paule‐Mandel estimator also coincides with this value when *n*=2 so that all 3 estimators satisfy *H* ^∗^=1 when ${\hat{\tau }}^{2}>0$ and *n*=2.

Writing the summation (2) explicitly as the sum of *n*=2 terms, using a common denominator of *w*_1_+*w*_2_ for all expressions inside the quadratic terms, and simplifying the resulting expression gives

(4) $$H^{*2}=\frac{w_{1}w_{2}{\left(y_{1}-y_{2}\right)}^{2}}{w_{1}+w_{2}}.$$

To estimate *τ* ^2^ using the Paule‐Mandel method, we set *H* ^∗2^=1. Setting (4) equal to 1 gives ${\left(y_{1}-y_{2}\right)}^{2}=1/w_{1}+1/w_{2}=\sigma_{1}^{2}+\sigma_{2}^{2}+2{\hat{\tau }}^{2}$ so that the Paule‐Mandel estimator is equal to the expression for the DerSimonian and Laird and REML estimators given by Rukhin. Hence, all 3 estimators satisfy *H* ^∗2^=1 whenever ${\hat{\tau }}^{2}>0$ and *n*=2, because all 3 estimators are then the same and the Paule‐Mandel estimator ensures this by construction. Therefore, groups 7 and 8 are impossible, because *H* ^∗2^=1 ensures that the modified method's 95% confidence interval is the widest. Group 9 has already been established to be impossible for the DerSimonian and Laird and Paule‐Mandel estimators, and Rukhin's demonstration of the equivalence between the REML and DerSimonian and Laird estimators when *n*=2 means that this group is also impossible when using REML in this setting.

Hence, for the special case where *n*=2, we have the Paule‐Mandel 3‐group taxonomy identified in Section 5.4.3 but it now applies to all 3 estimators. The 3‐group taxonomy consists of group “1 or 5,” 13, and 16 as shown in Table 2. Since *H* ^∗^=1 when ${\hat{\tau }}^{2}>0$ for this special case, all methods that use the modification when ${\hat{\tau }}^{2}>0$ are tabulated as “RE(t)” for group 1 or 5, because the modified method's standard error is the same as the conventional one when *H* ^∗^=1. None of the 3 groups provide *H* ^∗^>1 in this special case, and so the role of *H* ^∗^ in the reduced taxonomy is omitted in Table 2.

**Table 2:** A taxonomy of meta‐analysis datasets as in Table 1 but for the much simpler special cases where n=2 or all studies are the same “size”

				Never	Always	Constrain	Hybrid 1	Hybrid 2	Hybrid 3
Group	${\hat{\tau }}^{2}>0$	Mod>RE	Mod>CE	(Section 4.1)	(Section 4.2)	(Section 4.3)	(Section 4.4)	(Section 4.5)	(Section 4.6)
1 or 5	Yes	Yes	Yes	RE(Z)	RE(t)	RE(t)	RE(t)	RE(t)	RE(t)
13	No	Yes	Yes	CE(Z)	Mod(t)	CE(t)	CE(Z)	Mod(t)	Mod(t)
16	No	No	No	CE(Z)	Mod(t)	CE(t)	CE(Z)	CE(Z)	CE(Z)

*H* ^∗^=1 when ${\hat{\tau }}^{2}>0$, so the modified standard error is the same as the standard one for group “1 or 5.” Hence, all methods that use the modification when ${\hat{\tau }}^{2}>0$ are tabulated as “RE(t)” for group 1 or 5.

### 5.6 The reduced taxonomy for the special case where all studies are the same “size”

We will also examine the special case where all studies are the same “size,” that is, $\sigma_{i}^{2}=\sigma^{2}$ for all *i*. This artificial special case has been used previously to give an indication of how methods for meta‐analysis perform.^5^, ^18^, ^33^ The mathematics is greatly simplified in this situation. In this special case, the DerSimonian and Laird, REML, and Paule‐Mandel estimators become equivalent and, before truncation, we have $\sigma^{2}+{\hat{\tau }}^{2}=s^{2}$, where *s* ^2^ is the sample variance.^18^, ^33^ This result is easily established for the DerSimonian and Laird and Paule‐Mandel estimators from equating $\sigma_{i}^{2}=\sigma^{2}$ in their estimating equations and using a little algebra, where we also note that the pooled estimators of *μ* that appear in the estimating equations are equal to $\bar{y}$. For REML, this result is similarly easily obtained from the expression for the restricted log likelihood given by Normand,^34^ her page 336, upon setting $\sigma_{i}^{2}=\sigma^{2}$, differentiating with respect to *τ* ^2^ and setting the resulting expression to 0. Hence, we have a second special case where all 3 estimators coincide.

Hence, if ${\hat{\tau }}^{2}>0$, then ${\hat{\tau }}^{2}=s^{2}-\sigma^{2}$. Furthermore $\hat{\mu }=\bar{y}$, where $\bar{y}$ is the average of the *Y*_*i*_. Equating ${\hat{\tau }}^{2}=s^{2}-\sigma^{2}$ and $\hat{\mu }=\bar{y}$ in (2) immediately yields *H* ^∗2^=1. This means that the distinction between groups 1 and 5 in Table 1 becomes meaningless, just as it is for the case where *n*=2. Furthermore, groups 7 and 8 are impossible when $\sigma_{i}^{2}=\sigma^{2}$ for all *i* because we have seen that ${\hat{\tau }}^{2}>0$ implies that *H* ^∗^=1, which ensures that the modified method's 95% confidence interval is the widest. Finally, group 9 is impossible in this special case because all 3 estimators reduce to the same estimator for this special case, and we have already established the impossibility of group 9 for both the DerSimonian and Laird and Paule‐Mandel estimators.

Hence, in this special case, the taxonomy of datasets in Table 1 becomes the same 3‐group taxonomy as for the *n*=2 case, as already shown in Table 2. This 3‐group taxonomy would therefore appear to be sufficient to give a reasonable guide to how the various methods for analysis compare, because it is likely to be adequate to describe small *n* scenarios (it describes the *n*=2 case) and because it is likely to be an adequate representation when study sizes are not very disparate (it describes the $\sigma_{i}^{2}=\sigma^{2}$ case). The analysis of these 2 special cases indicates that we can anticipate that meta‐analyses in groups 7, 8, and 9 will be rare so that the other 4 groups in Table 1 will be the most important when determining the implications of using each of the 6 analysis methods. This will be confirmed in our empirical investigation, and so we suggest that the reduced taxonomy in Table 2 will often be adequate to obtain a guide to the implications of using each method. We will use the full taxonomy in our empirical investigation, but the reduced taxonomy greatly simplifies the assessment of the likely implications of analyses based on the modified method. Applied analysts may therefore prefer to justify their choice of analysis using the reduced, rather than the full, taxonomy.

## 6 EMPIRICAL INVESTIGATION

We performed an empirical investigation to further examine the implications of using the 6 analysis methods. For our empirical investigation we use binary outcome data from meta‐analyses included in the Cochrane Database of Systematic Reviews (Issue 1, 2008), which were provided to us by the Nordic Cochrane Centre. We use the log odds ratio as the measure of treatment effect, where halves were added to all entries in tables that contain zeroes. Most Cochrane reviews contain multiple meta‐analyses, corresponding to different pairwise comparisons of interventions and different outcomes examined. Davey et al 35 classified each meta‐analysis by outcome type, the type of interventions compared, and the medical specialty. Here, we use data on the first reported binary outcome meta‐analysis within each of the 1991 Cochrane reviews reporting at least one binary outcome meta‐analysis in the full database extracted by Davey et al.^35^

However, we removed 13 of these meta‐analyses from our investigation. Seven were removed because they involve 2 studies that provide exactly the same estimated treatment effects; this is a theoretical impossibility under the random‐effects model but can occur in numerical data and causes immediate problems for the modified method. This is because then *H* ^∗^=0 so that the modified method's standard error is zero. Since it is difficult to ascertain what applied meta‐analysts might do in such instances, these meta‐analyses were removed. For 6 other meta‐analyses, the REML estimator of *τ* ^2^ failed to converge using the *metafor* default settings; although it was possible, with effort, to force convergence in these instances, it is again unclear how applied meta‐analysts might proceed in such instances. This leaves us with 1978 meta‐analyses in our empirical investigation.

There is then the difficulty that many of the remaining meta‐analyses have *n*=2 studies. As explained above, the full taxonomy is not possible for these datasets. We therefore initially restricted our investigation to those meta‐analyses that have *n*⩾3 so that the full taxonomy is possible and the results are shown in Table 3. As explained above, groups 7, 8, and 9 are impossible if the Paule‐Mandel estimator is used, and group 9 is impossible if the DerSimonian and Laird estimator is used; these entries are shown with a “X” in Table 3. Furthermore, for the Paule‐Mandel estimator, the distinction between groups 1 and 5 is meaningless; all meta‐analyses where ${\hat{\tau }}^{2}>0$ are denoted as group 1 in Table 3, and group 5 is denoted as a “X.” The estimator of *τ* ^2^ does not greatly change the overall conclusions: Noting that for the Paule‐Mandel estimator groups 1 and 5 are essentially one group, the groups that meta‐analyses are allocated to are in agreement 1366/1376 times (for the DerSimonian and Laird and Paule‐Mandel estimators), 1221/1376 times (REML and Paule‐Mandel estimators), and 1179/1376 times (DerSimonian and Laird and REML estimators). For the remaining *n*=2 meta‐analyses, all estimators are the same and 220/602 (36.5%) meta‐analyses belong to groups 1 or 5, 310/602 (51.5%) belong to group 13, and 72/602 (12.0%) belong to group 16.

**Table 3:** Empirical investigation: the number and percentage of meta‐analyses that belong to each group in the taxonomy shown in Table 1 for n⩾3

Estimator	Group 1	Group 5	Group 7	Group 8	Group 9	Group 13	Group 16
DerSimonian and Laird	422 (30.7%)	296 (21.5%)	10 (0.7%)	0 (0%)	X	318 (23.1%)	330 (24.0%)
REML	378 (27.5%)	410 (29.8%)	39 (2.8%)	15 (1.1%)	10 (0.7%)	215 (15.6%)	309 (22.5%)
Paule‐Mandel	728 (52.9%)	X	X	X	X	318 (23.1%)	330 (24.0%)

For the DerSimonian and Laird estimator, group 9 is impossible. For the Paule Mandel estimator, groups 7, 8, and 9 are impossible, and the distinction between groups 1 and 5 is meaningless; all meta‐analyses where the Paule‐Mandel estimator is positive are denoted as group 1. “X” denotes that the group is impossible for the estimator used, subject to the caveat that for the Paule‐Mandel estimator groups 1 and 5 are essentially a single group.

The reader is encouraged to examine the proportions of meta‐analyses in each group in this empirical investigation (Table 3), and also to consider the type of meta‐analysis that their preferred method produces (Table 1), to assess the likely implications of their approach. Of interest is that no meta‐analysis has been observed in group 8 when the DerSimonian and Laird estimator is used. We conclude therefore that it is extremely unlikely that the modified method provides shorter 95% confidence intervals than the conventional common‐effect model when the DerSimonian and Laird estimator is positive. This observation is a weak type of transposition of the second potential concern in Section 3.2. However, we have simulated an example of a meta‐analysis that belongs to group 8 when using the DerSimonian and Laird estimator, to establish that this is possible. We note that the Paule‐Mandel and DerSimonian and Laird estimators allocate meta‐analysis datasets to the same group more often than the other 2 combinations of estimators. The empirical investigation suggests that this is because REML has a slight tendency to produce fewer meta‐analyses in group 13 and more meta‐analyses in groups 7, 8, and 9 (and group 7 in particular, although these groups remain rare). This suggests that REML may have a slightly greater tendency to produce positive estimates of *τ* ^2^ whilst resulting in modified confidence intervals that are shorter than the more conventional one.

One important observation from Table 1 is that the groups where hybrid methods 2 and 3 result in different analyses (groups 7 and 8) are in general quite rare. Recall that hybrid method 3 was motivated by the recommendation of Wiksten et al,^27^ who suggest presenting a sensitivity analysis using the common‐effect model. Hybrid method 2 can therefore be motivated by the alternative recommendation of presenting a sensitivity analysis using the standard procedure for random‐effects meta‐analysis. Our empirical investigation indicates that hybrid methods 2 and 3 will very often result in the same analysis, and our reduced taxonomy (Table 2) supports this conclusion. On the basis that hybrid method 2 is likely to be preferable to analysts who would prefer to avoid using the fixed‐effect model, we therefore suggest an amendment to the recommendation of Wiksten et al 27: we instead recommend that a conventional random‐effects meta‐analysis is performed as a sensitivity analysis when using the modified method.

We can also use this empirical investigation to directly address concerns 2 and 3. We have established that groups 7 and 8 are quite rare, so it is unusual for the modified method to result in shorter confidence intervals than the standard method when ${\hat{\tau }}^{2}>0$. Group 16 is however quite common, which indicates that it is quite common for the modified method to result in shorter confidence intervals when ${\hat{\tau }}^{2}=0$. Concern 2 is therefore genuine but is most likely to apply when ${\hat{\tau }}^{2}=0$. However, group 13 is also quite common, and the overall impression is that the modified method is as likely to result in longer, rather than shorter, confidence intervals when ${\hat{\tau }}^{2}=0$. This indicates that inference conditional on ${\hat{\tau }}^{2}=0$ using the modified method will not compare badly to the standard method; this conclusion is supported by simulation studies performed by the second author that may form the subject of future work. We conclude that concern 3 is an illusion. Simulation studies such as these could also be used to examine concern 1 in detail, and this too may be the subject of future work. The general impression from the taxonomy in Table 1, and the empirical rates in Table 3, is that the way in which the modified method is used can have important implications for the resulting analysis. Concern 4 would therefore also seem to be genuine.

To summarise our findings, we conclude that there are some residual concerns when using the modified method in practice. Entirely abandoning the standard method would therefore seem to us to be, at best, premature. Despite this, because simulation studies have consistently found that the modified method performs well,^7^, ^8^, ^9^, ^12^ the case for it replacing the standard method as the default remains strong.

## 7 DISCUSSION

We have explored the implications of using the modified method for random‐effects meta‐analysis. Further investigation would be welcome, but we align ourselves with those who argue that the modified method should now be given a fundamental role in standard meta‐analysis methodologies. This is because simulation studies have repeatedly demonstrated that the modified method performs well. We therefore suggest that groups such as Cochrane should consider using it in conjunction with more standard approaches. Despite this, analysts who advocate the modified method should be ready to defend its use against the possible objections to it that we have presented. The most serious, and quite commonly encountered, genuine concern about the modified method that we have been able to identify is that it can provide shorter confidence intervals, and smaller *P* values, than standard methods. This is a serious issue because the usual concern is that these standard methods are themselves anticonservative. We therefore advocate sensitivity analyses along the lines proposed by Wiksten et al when using the modified method. We have however determined that, in practice, it is unimportant whether a common‐effect or a conventional random‐effect analysis is used as a sensitivity analysis when using the modified method. Hence, we recommend amending the recommendation by Wiksten et al; we suggest instead using a conventional random‐effects analysis for the sensitivity analysis. This is based on our preference for random‐effects models. However, the main point would still seem to perform some form of sensitivity analysis when using the modified method, especially when the data appear to be homogenous. This is because if *H* ^∗^ is very small, then artificially small standard errors, short confidence intervals, and small *P* values could be obtained when using the modified approach. Hence, we seek here to embellish the important work and main conclusion of Wiksten et al, rather than detract from this.

One important contribution of this paper has been to create a new taxonomy of meta‐analysis datasets. This provides a conceptual framework in which the implications of the modified method can be assessed. Our hope is that our work will allow applied analysts to make a more informed decision about whether or not the modified method should replace the standard method as the default and also that this paper will further stimulate discussion relating to this issue. For example, the taxonomy shows that the implications of using the modified approach are different when alternative estimators of the between‐study variance are used, although our empirical investigation suggests that the choice of this estimator does not greatly change the main conclusions. Our new framework enables us to determine the implications of using the modified approach more clearly and concretely than was previously possible. However, further refinement of the taxonomy is both possible and desirable. For example, the taxonomy only identifies whether or not the modified method results in wider confidence intervals than standard methods. It does not attempt to quantify the extent to which the lengths of modified confidence intervals differ with conventional ones, and therefore whether or not particular methods may have performed poorly to the extent that they are misleading. Our taxonomy was produced with the less ambitious aim to determine whether or not alternative conventions result in different analyses being presented, rather than if qualitatively different conclusions are made. Extending the taxonomy, so that it contains groups that depend on the extent to which the modified and standard methods differ, is an obvious next step. However, this type of extension faces immediate challenges. This is because we must then determine additional thresholds, for example, to determine whether or not the modified method provides a “notably shorter” confidence interval. Achieving a consensus about what constitutes “notably shorter” will, however, be extremely difficult in practice. Another issue is that more ambitious taxonomies such as this will result in more groups so that much larger empirical investigations will be needed to adequately explore them. Despite this, forming taxonomies that explore a wider range of possibilities, with the aim of identifying when the modified and standard methods result in markedly different, rather than just nonidentical, results is an important avenue for further research. We encourage those with very large databases to consider this possibility. This type of investigation could begin by using the taxonomy that we have proposed and then introducing more specific criteria that distinguish between inferences from meta‐analyses that are deemed to be different to a greater, or lesser, extent.

To summarise, we have developed a new conceptual framework to evaluate the modified method for random‐effects meta‐analysis. We have investigated the implications of using the modified method empirically and have also examined some closely related variants of it. We have suggested an amendment to a recently made recommendation. We are confident that our amended recommendation will be more acceptable to those who favour the random‐effects model over the common‐effect model.

## References

[1] Higgins JPT , Thompson SG , Spiegelhalter DJ . A re‐evaluation of random‐effects meta‐analysis. J R Stat Soc Ser A. 2009;172:137‐159. 10.1111/j.1467-985X.2008.00552.x PMC2667312 19381330

[2] Ades AE , Lu G , Higgins JPT . The interpretation of random‐effects meta‐analysis in decision models. Med Decis Making. 2005;25:646‐654. 16282215 10.1177/0272989X05282643

[3] DerSimonian R , Laird N . Meta‐analysis in clinical trials. Controlled Clin Trials. 1986;7:177‐188. 3802833 10.1016/0197-2456(86)90046-2

[4] Jackson D , Bowden J , Baker R . Approximate confidence intervals for moment‐based estimators of the between‐study variance in random effects meta‐analysis. Res Synth Methods. 2015;6:372‐382. 26287958 10.1002/jrsm.1162 PMC4839498

[5] Jackson D , Bowden J , Baker R . Hoes does the DerSimonian and Laird procedure for random effects meta‐analysis compare with its more efficient but harder to compute counterparts? J Stat Plann Inference. 2010;140:961‐970.

[6] Hartung J . An alternative method for meta‐analysis. Biometrical J. 1999;41:901‐916.

[7] Hartung J , Knapp G . On tests of the overall treatment effect in meta‐analysis with normally distributed responses. Stat Med. 2001;20:1771‐1782. 11406840 10.1002/sim.791

[8] Hartung J , Knapp G . A refined method for the meta‐analysis of controlled clinical trials with binary outcome. Stat Med. 2001;20:3875‐3889. 11782040 10.1002/sim.1009

[9] Sidik K , Jonkman JN . A simple confidence interval for meta‐analysis. Stat Med. 2002;21:3153‐3159. 12375296 10.1002/sim.1262

[10] Copas J . Letters to the editor. Stat Med. 2003;22:2667‐2668. 12898551 10.1002/sim.1549

[11] Sidik K , Jonkman JN . Authors reply. Stat Med. 2004;23:159‐162.

[12] IntHout J , Ioannidis JPA , Borm GF . The Hartung‐Knapp‐Sidik‐Jonkman method for random effects meta‐analysis is straightforward and considerably outperforms the standard DerSimonian‐Laird method. BMC Med Research Method. 2014;14:25. 10.1186/1471-2288-14-25 PMC4015721 24548571

[13] Röver C , Knapp G , Friede T . Hartung‐Knapp‐Sidik‐Jonkman approach and its modification for random‐effects meta‐analysis with few studies. BMC Med Res Method. 2015;15:99. 10.1186/s12874-015-0091-1 PMC4647507 26573817

[14] Viechtbauer W . Conducting meta‐analyses in R with the metafor package. J Stat Software. 2010;36:1‐48.

[15] Schwarzer G . meta: An R package for meta‐analysis. R News. 2007;7:40‐45.

[16] Knapp G , Hartung J . Improved tests for a random effects meta‐regression with a single covariate. Stat Med. 2003;22:2293‐2710. 10.1002/sim.1482 12939780

[17] Jackson D , Riley RD . A refined method for multivariate meta‐analysis and meta‐regression. Stat Med. 2014;33:541‐554. 23996351 10.1002/sim.5957 PMC4285306

[18] Jackson D , Bowden J . A re‐evaluation of the ‘quantile approximation method’ for random effects meta‐analysis. Stat Med. 2009;28:338‐348. 19016302 10.1002/sim.3487 PMC2991773

[19] Noma H . Confidence intervals for a random‐effects meta‐analysis based on Bartlett‐type corrections. Stat Med. 2011;30:3304‐3312. 21964669 10.1002/sim.4350

[20] Baker R , Jackson D . Meta‐analysis inside and outside particle physics: two traditions that should converge? Res Synth Methods. 2013;4:109‐124. 26053651 10.1002/jrsm.1065

[21] Jackson D , Baker R . Meta‐analysis inside and outside particle physics: convergence using the path of least resistance? Res Synth Methods. 2013;4:125‐126. 26053652 10.1002/jrsm.1079

[22] Veroniki AA , Jackson D , Viechtbauer W , et al. Methods to estimate the between‐study variance and its uncertainty in meta‐analysis. Res Synth Methods. 2016;7:55‐79. 26332144 10.1002/jrsm.1164 PMC4950030

[23] Higgins JPT , Thompson SG . Quantifying heterogeneity in a meta‐analysis. Stat Med. 2002;21:1539‐1558. 12111919 10.1002/sim.1186

[24] Knapp G , Biggerstaff BJ , Harting J . Assessing the amount of heterogeneity in random‐effects meta‐analysis. Biometrical J. 2006;48:271‐285. 10.1002/bimj.200510175 16708778

[25] Viechtbauer W . Confidence intervals for the amount of heterogeneity in meta‐analysis. Stat Med. 2007;26:37‐52. 16463355 10.1002/sim.2514

[26] Hoaglin D . Misunderstandings about Q and ‘Cochran's Q test' in meta‐analysis. Stat Med. 2016;35:485‐495. 26303773 10.1002/sim.6632

[27] Wiksten A , Rücker G , Schwarzer G . Hartung‐Knapp method is not always conservative compared to fixed effect meta‐analysis. Stat Med. 2016;35:2503‐2515. 26842654 10.1002/sim.6879

[28] Turner RM , Davey J , Clarke MJ , Thompson SG , Higgins JP . Predicting the extent of heterogeneity in meta‐analysis, using empirical data from the Cochrane Database of Systematic Reviews. Int J Epidemiol. 2012;41:818‐827. 22461129 10.1093/ije/dys041 PMC3396310

[29] Casella G . Conditional inference from confidence sets Current Issues in Statistical Inference: Essays in Honor of D. Basu, Lecture Notes‐Monograph Series, vol. 17. California: Institute of Mathematical Statistics; 1992:1‐12.

[30] Poole C , Greenland S . Random‐effects meta‐analyses are not always conservative. Am J Epidemiol. 1999;150:1‐12. 10472946 10.1093/oxfordjournals.aje.a010035

[31] Paule RC , Mandel J . Concensus values and weighting factors. J Res Nat Bur Stand. 1982;87:377‐385. 10.6028/jres.087.022 PMC6768160 34566088

[32] Rukhin AL . Estimating common mean and heterogeneity variance in two study case meta‐analysis. Stat Probab Lett. 2012;82:1318‐1325.

[33] Jackson D . The significance level of the standard test for a treatment effect in meta‐analysis. Stat Biopharm Res. 2009;1:92‐100.

[34] Normand SL . Tutorial in Biostatistics. Meta‐analysis: formulating, evaluating, combining and reporting. Stat Med. 1999;18:321‐359. 10070677 10.1002/(sici)1097-0258(19990215)18:3<321::aid-sim28>3.0.co;2-p

[35] Davey J , Turner RM , Clarke MJ , Higgins JPT . Characteristics of meta‐analyses and their component studies in the Cochrane Database of Systematic Reviews: a cross‐sectional, descriptive analysis. BMC Med Res Method. 2011;11:160. 10.1186/1471-2288-11-160 PMC3247075 22114982

